# Trogocytosis of neurons and glial cells by microglia in a healthy adult macaque retina

**DOI:** 10.1038/s41598-023-27453-2

**Published:** 2023-01-12

**Authors:** Megan Goyal, Andrea S. Bordt, Jay Neitz, David W. Marshak

**Affiliations:** 1 Department of Neurobiology and Anatomy, McGovern Medical School, Houston, TX USA; 2 Department of Ophthalmology, University of Washington, Seattle, WA USA

**Keywords:** Glial biology, Visual system

## Abstract

Microglial cells are the primary resident immune cells in the retina. In healthy adults, they are ramified; that is, they have extensive processes that move continually. In adult retinas, microglia maintain the normal structure and function of neurons and other glial cells, but the mechanism underlying this process is not well-understood. In the mouse hippocampus, microglia engulf small pieces of axons and presynaptic terminals via a process called trogocytosis. Here we report that microglia in the adult macaque retina also engulf pieces of neurons and glial cells, but not at sites of synapses. We analyzed microglia in a volume of serial, ultrathin sections of central macaque retina in which many neurons that ramify in the inner plexiform layer (IPL) had been reconstructed previously. We surveyed the IPL and identified the somas of microglia by their small size and scant cytoplasm. We then reconstructed the microglia and studied their interactions with other cells. We found that ramified microglia frequently ingested small pieces of each major type of inner retinal neuron and Müller glial cells via trogocytosis. There were a few instances where the interactions took place near synapses, but the synapses, themselves, were never engulfed. If trogocytosis by retinal microglia plays a role in synaptic remodeling, it was not apparent from the ultrastructure. Instead, we propose that trogocytosis enables these microglia to present antigens derived from normal inner retinal cells and, when activated, they would promote antigen-specific tolerance.

## Introduction

Microglia are the principal type of immune cell in the retina. During development, they have both positive and negative effects on retinal neurons mediated by the release of cytokines. Some microglial cytokines promote proliferation and survival of retinal neurons and others contribute to programmed cell death. In addition to these trophic effects, retinal microglia also remove dying neurons via phagocytosis^1^. In the developing brain, microglia also influence the growth and positioning of neurons, the growth of neurites and the formation of synapses. Microglia also eliminate excess synapses in the developing brain^2^. It is uncertain whether microglia influence the formation or maintenance of synapses during development of the retina^1^. In adults, retinal microglia become activated and assume an amoeboid morphology in response to infection, injury and other insults, including hypoxia, neurodegenerative diseases and many eye diseases^3,4^.

Less is known about the roles of microglia in the homeostasis of healthy adult retinas, where they have a ramified morphology and are located in the plexiform layers^5,6^. In adult mouse retinas, processes of microglia retract and extend at a rate of approximately 5 µm/min. In addition, new processes form and others are eliminated^7,8^. Ionotropic glutamatergic neurotransmission potentiates this motility and ionotropic GABAergic transmission inhibits it. Both effects are mediated indirectly via extracellular ATP^9^. Microglia are essential to maintain retinal function in healthy adult animals. In adult mice whose microglia had been ablated, synapses made by cone pedicles, rod bipolar cell axon terminals and amacrine cell processes developed abnormal ultrastructure, and the amplitude of the b-wave of the electroretinogram was reduced^10^. It is not clear how microglia in the normal adult retina maintain the integrity of these synapses. In order to investigate this, we described the interactions of microglia with neurons and Müller glial cells of the inner retina of adult macaque retina at the ultrastructural level.

## Methods

### Electron microscopy

Retinal tissue was obtained from a healthy, 9 year-old male macaque (Macaca nemestrina) through the Tissue Distribution Program at the Washington National Primate Center. All procedures were approved by the Institutional Animal Care and Use Committee at the University of Washington. Central retinal tissue was processed for serial blockface scanning electron microscopy (SBFSEM) as previously described^11^. Briefly, a 1 × 1 mm square block of central retina was fixed in glutaraldehyde, stained en bloc with osmium ferrocyanide, uranyl acetate and lead aspartate and then embedded in epoxy resin. The selected area, located approximately 2 mm temporal to the center of the fovea, was particularly well-suited for connectomic analysis because the neurons were relatively small. The images were acquired using a Zeiss Sigma VP field emission scanning electron microscope equipped with a 3View system (Gatan, Inc.).

### Connectomic analysis

The retinal volume was sectioned at a thickness of 70 nm in the horizontal plane and images were acquired at a resolution of 7.5 nm/pixel. This volume was also used in recent studies of synaptic inputs to retinal ganglion cells^12,13^. Image registration was performed using Nornir (http://nornir.github.io RRID:SCR_003584), and the image tiles were reassembled into cohesive digital volumes and hosted on a 24-core server at the University of Washington.

The serial EM volume was annotated using the web-based, multiuser Viking software described previously^14^ (RRID:SCR_005986). Briefly, profiles of processes were typically annotated by placing circular discs with the same diameter at their centers of mass and linking them to annotations on adjacent sections. In some instances, cells were annotated with closed curves in order to provide more realistic renditions. Sites of trogocytosis were annotated and linked to the microglia in which they were located. Neurons, glial cells and other structures were numbered consecutively. Because of the en bloc staining that is required for blockface imaging, unspecialized plasma membranes are more electron-dense, and the synaptic ribbons and synaptic densities are not as prominent as they would be in transmission electron microscopy.

The major neuronal and glial cell types were identified using ultrastructural criteria^15–17^. Axon terminals of bipolar cells contained numerous synaptic vesicles and synaptic ribbons. Because the contrast of the synaptic ribbons in this volume is not as high as in images from transmission electron microscopy, we confirmed the identity of the presynaptic bipolar cells by reconstructing the axon terminals and, whenever possible, the somas and primary dendrites. Axons and dendrites of amacrine cells contained fewer synaptic vesicles, and they were typically clustered at synapses. Retinal ganglion cells had somas in the ganglion cell layer (GCL) and axons in the optic fiber layer; their dendrites were exclusively postsynaptic. Processes of Müller glial cells were identified by their orientation parallel to the plane of the retina, high cytoplasmic electron density and abundant glycogen granules. The somas of microglial cells in the IPL were small and elongated; they had very little perinuclear cytoplasm and contained many lysosomes.

### Data analysis

Data analysis and three dimensional rendering were performed using an open-source Matlab (Mathworks, RRID: SCR_001622) program https://github.com/neitzlab/sbfsem-tools RRID: SCR_017350. The image rendering was performed using the RenderApp function^18^. Using the SynapseSphere function, sites of trogocytosis were rendered as unit spheres centered at its X, Y and Z coordinates then scaled to optimize visibility. Processes of microglia and neurons were analyzed using the IPLDepth function^12^. The boundary between the inner nuclear layer (INL) and the inner plexiform layer (IPL) was designated as 0% and the IPL-GCL boundary as 100% depth. For descriptive purposes, the IPL was divided into 5 strata (S) of 20% each with S1 adjacent to the INL and S5 adjacent to the GCL. The geometric distances between the sites of trogocytosis and the synaptic densities were calculated using Microsoft Excel.

## Results

**Figure 1:**
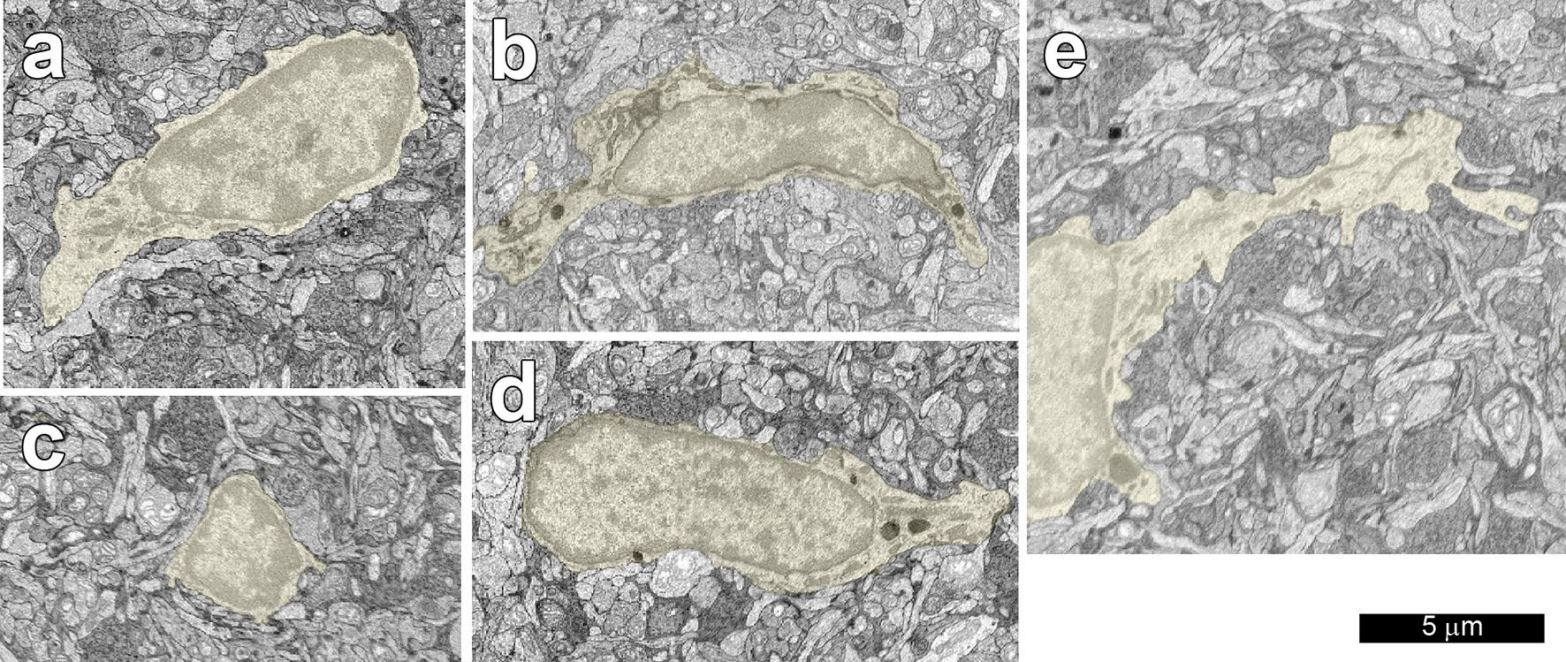
Microglial cell somas (yellow): (**a**) 50688, (**b**) 50640, (**c**) 50636, (**d**) 1 and (**e**) 50635. Note that a portion of the soma of cell 50635 (**e**) is not included in the volume analyzed. All of the somas were small and elongated. The shape is not apparent in the horizontal plane of section for cell 50636, but see Fig. 3.

The somas of microglial cells in the IPL were clearly distinguishable from those of other types of cells (Fig. 1). Somas of some types of amacrine cells are located in the IPL, but the two types of cells could be easily distinguished. Microglial cell somas were considerably smaller than those of amacrine cells, and their relatively short processes had many lysosomes but no synaptic specializations. Compared with the somas of neurons, the somas of microglial cells were elongated and contained very little cytoplasm.

**Figure 2:**
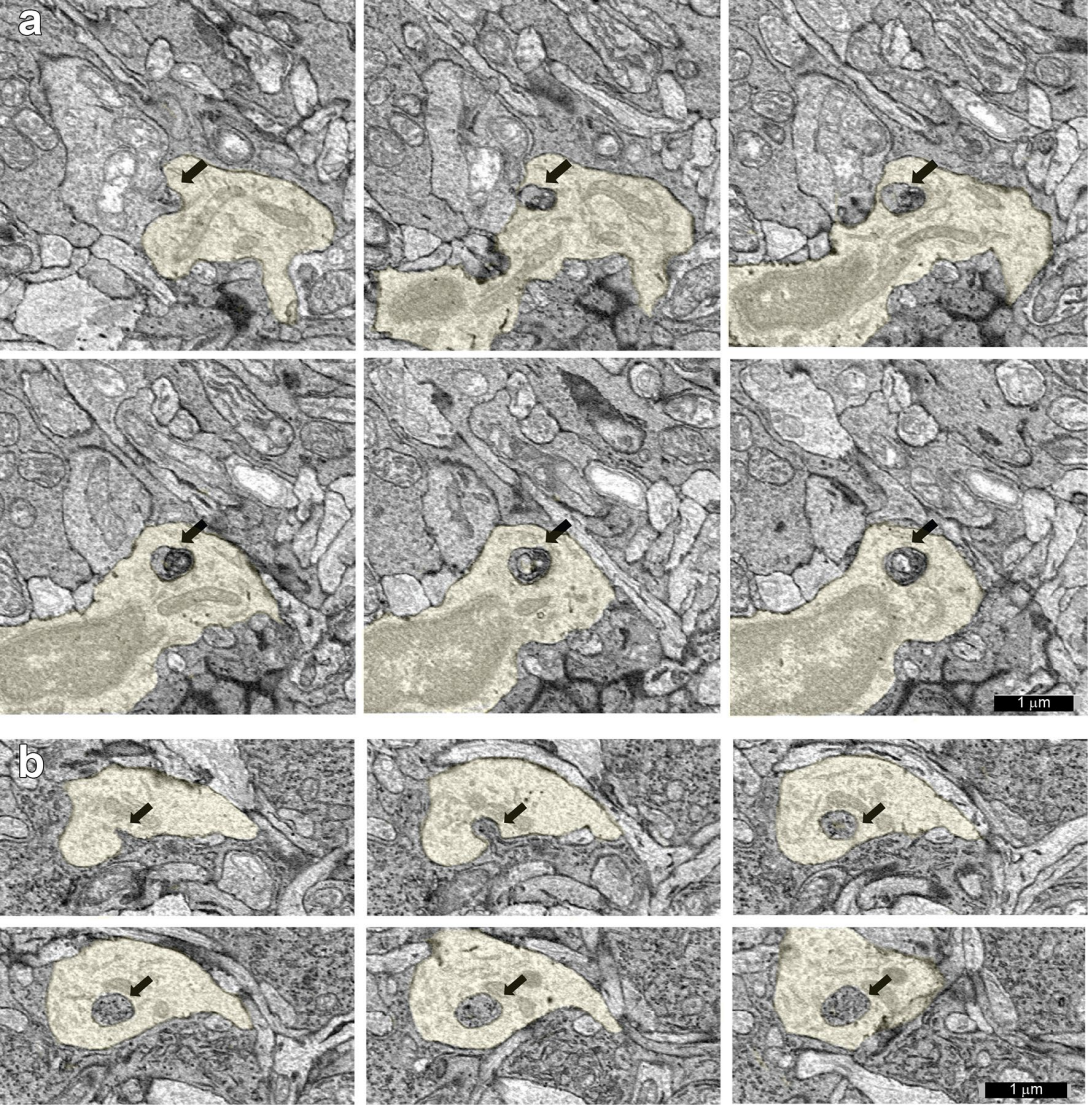
Examples of trogocytosis by processes of microglial cell #50688 (yellow). The ingested material is indicated with an arrow. (**a**) Trogocytosis of a neuron by the microglial cell, location ID: 1305081. (**b**) Trogocytosis of a Müller glial cell by the microglial cell, location ID: 1304768.

When a microglial cell engulfed a small piece of a neuron or a glial cell and a lysosome containing the fragment was observed in at least two adjacent sections, the interaction was classified as trogocytosis^19^. Figure 2 shows examples of trogocytosis of a neuron and a glial cell by one of the ramified microglial cells. This process is distinct from the phagocytosis of dead and dying cells by activated, amoeboid microglia. In phagocytosis, the ingested pieces are much larger and the underlying molecular mechanism is different^20^. There were lysosomes in the microglia that appeared to contain pieces of other cells but were no longer attached to those cells; these were not counted. There were many instances in which neurons simply indented the membrane of the microglial cells, and these were also not counted. In all, 5 ramified microglia and the 94 neurons and glial cells they interacted with were reconstructed (Table 1).

**Table 1:** Trogocytosis of macaque retinal neurons and Müller glial cells by microglia.

Microglial cell	Ganglion cells	Bipolar cells	Amacrine cells	Neuronal fragments*	Glial cells	Total
1	5	1	6	2	6	20
50635	4	3	7	2	8	24
50636	3	2	13	0	9	27
50688	3	3	5	2	3	16
50640	1	0	6	0	0	7
Total	16	9	37	6	26	94

The types of neurons and the Müller glial cells undergoing trogocytosis by each microglial cell are listed. In some instances, the neurons could not be reconstructed sufficiently to identify them with confidence, and these are called fragments.

**Figure 3:**
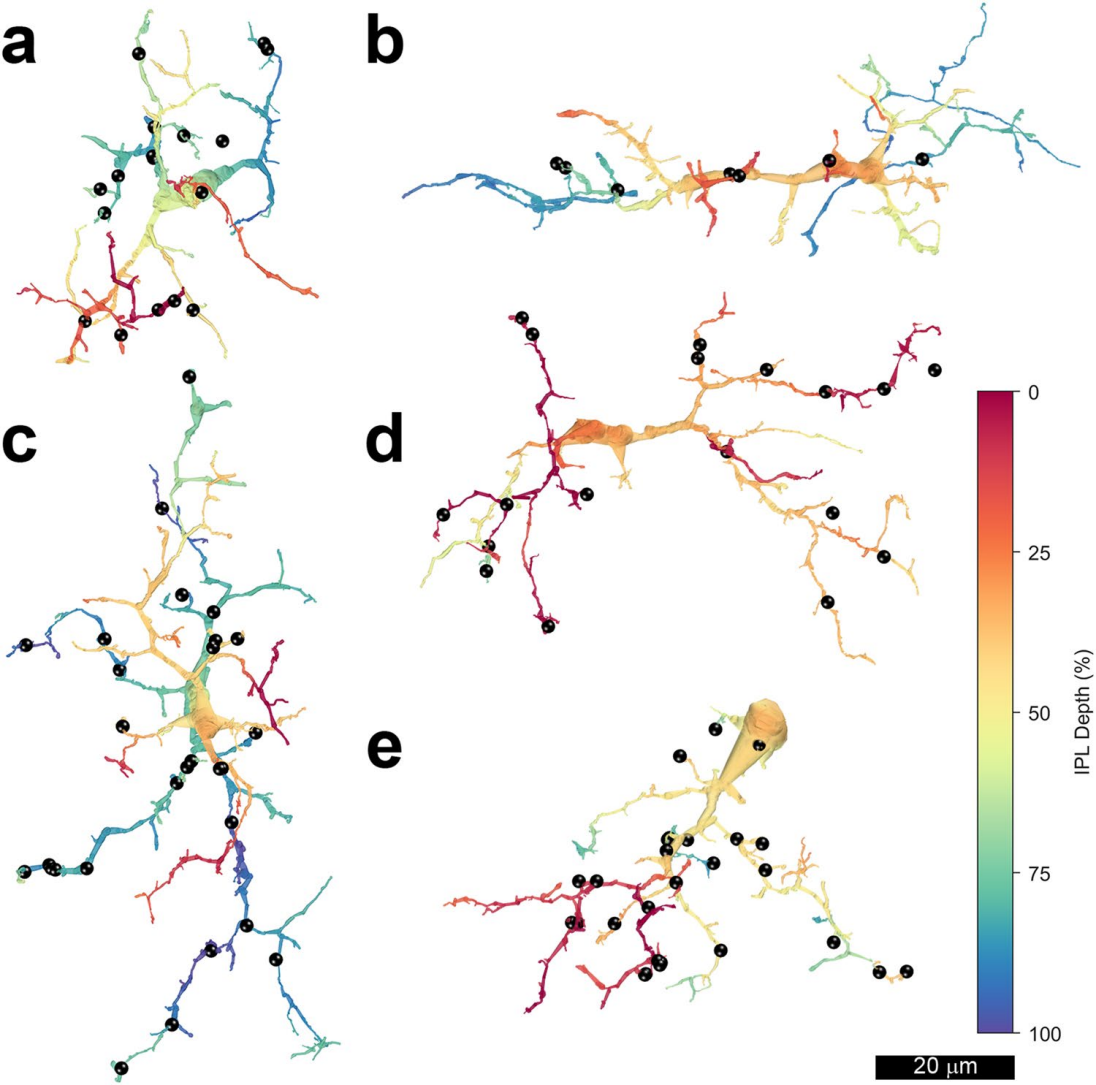
Renders of individual of microglial cells: (**a**) 50688, (**b**) 50640, (**c**) 50636, (**d**) 1 and (**e**) 50635. The depth of processes in the inner plexiform layer is indicated by pseudocolors; 0% (red) is the inner nuclear layer and 100% (blue) is the ganglion cell layer. Locations of trogocytosis are indicated (●). Note that the 5 microglia vary in the numbers and distribution of the sites of trogocytosis.

The reconstructed microglial cells were morphologically similar to the ramified microglia labeled with antibodies to ionized calcium binding adaptor protein 1 (Iba1) in the IPL of central macaque retina previously^6^. The 5 microglia and the sites of trogocytosis of neurons and glial cells are shown in Fig. 3. Compared with neurons, the microglia had irregularly shaped somas, thick primary processes and many short higher-order processes. The microglia varied in the orientation of their long axes.

The microglia also varied in the stratification of their processes, which are indicated by pseudocolors in Fig. 3. Three of the microglia had processes ramifying throughout the IPL, but their processes were not distributed uniformly. Cell 50688 had fewer processes in S2, and cells 50636 and 50640 had most of their processes in S3 and S5. The other two microglia had processes that did not reach all the strata of the IPL. Cell 1 had most of its processes in S1–S3, very few in S4 and none in S5. At least half of cell 50635 was not contained within the volume analyzed. Assuming it was relatively symmetrical, it would ramify in nearly all strata of the IPL with most of its processes in S1 and S3.

There was considerable variability in the number of neuronal and glial processes trogocytosed by the microglia. Even though only half of cell 50635 was contained in the volume, it trogocytosed pieces of 24 cells. This was nearly as many as cell 50636, which was contained entirely within the volume and trogocytosed 27 pieces. Microglial cell 1 trogocytosed 20 pieces of neurons and glia. These three microglia were similar to one another in the proportion of their interactions with glial cells; these comprised approximately one-third of the total. Fewer process were trogocytosed by the other two microglial cells: 16 by cell 50688 and 7 by cell 50640. These microglial cells also had a smaller proportion of their interactions with glia, 19% for cell 50688, and none at all for cell 50640.

**Figure 4:**
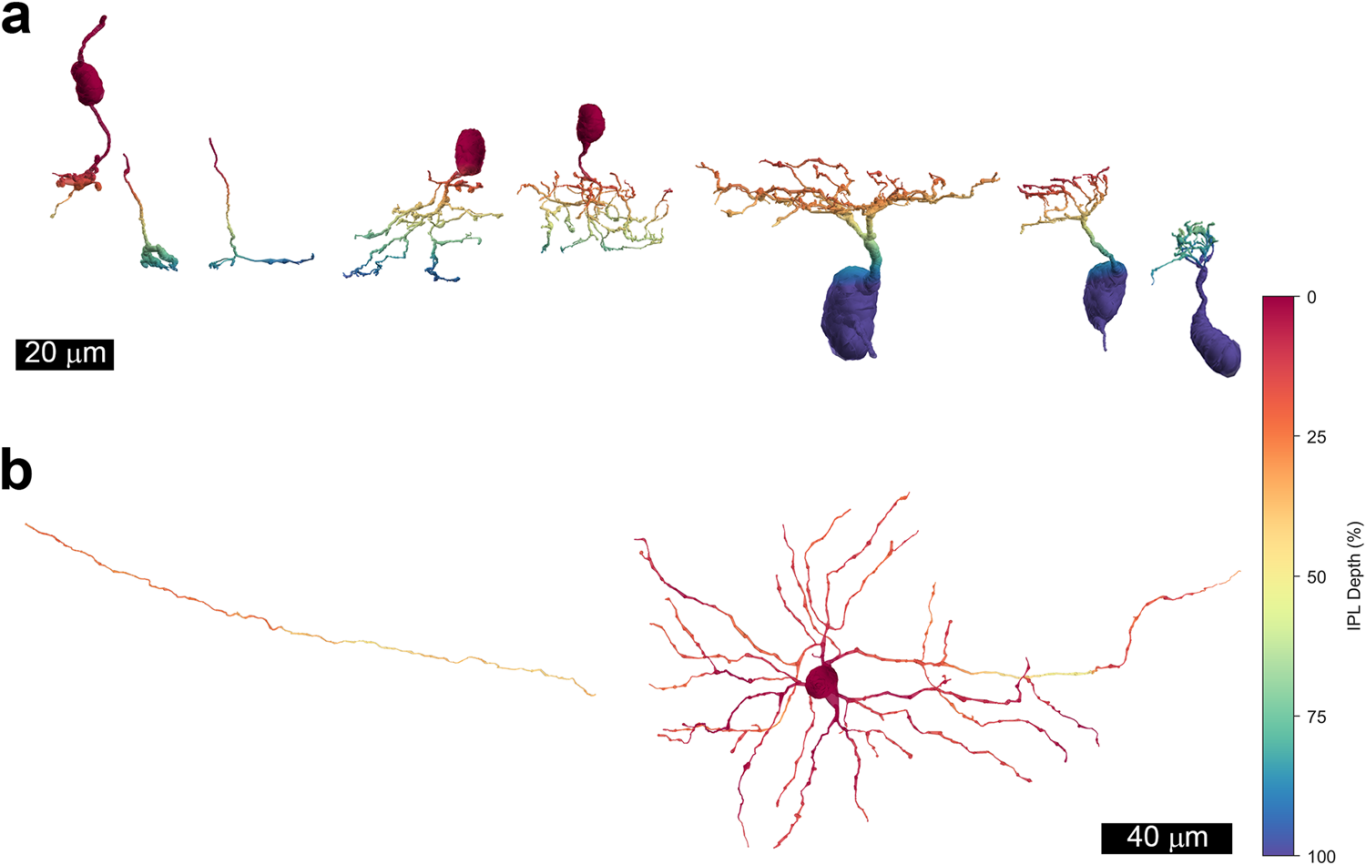
Examples of cells undergoing trogocytosis by microglial cells. The depth of processes in the inner plexiform layer is indicated by pseudocolors; 0% (red) is the inner nuclear layer and 100% (blue) is the ganglion cell layer. (**a**) Renders of individual cells as they would appear in vertical sections. From left to right: 44527 OFF Midget bipolar, 50942 ON Midget bipolar, 50955 S-ON bipolar, 50759 AII amacrine, 50594 knotty type 2 amacrine, 60963 OFF Parasol ganglion cell, 60957 OFF Midget GC and 50923 ON Midget GC. (**b**) Renders of individual cells as they would appear in flatmounts: 50917 a wide-field amacrine cell process and 50760 a medium-field amacrine cell narrowly stratified in S1 of the IPL.

The microglial cells trogocytosed pieces of all three major types of neurons ramifying in the IPL, and a number of these were identified morphologically. Ten of these neurons are illustrated in Fig. 4. The cells amacrine cells trogocytosed included narrow-field types knotty type 2 and AII^21,22^. One medium-field amacrine cell that was trogocytosed resembled the neuropeptide Y-immunoreactive cells described previously in macaque retina^23^. Several processes of wide-field amacrine cells were also trogocytosed, but these could not be identified because their somas were not contained within the volume analyzed. Retinal ganglion cell dendrites were also trogocytosed. These were from two of the major types of retinal ganglion cells, midget cells and parasol cells, and both ON and OFF subtypes were included. Bipolar cell axon terminals that were trogocytosed included those of midget, diffuse and blue cone bipolar cells^21^.

**Figure 5:**
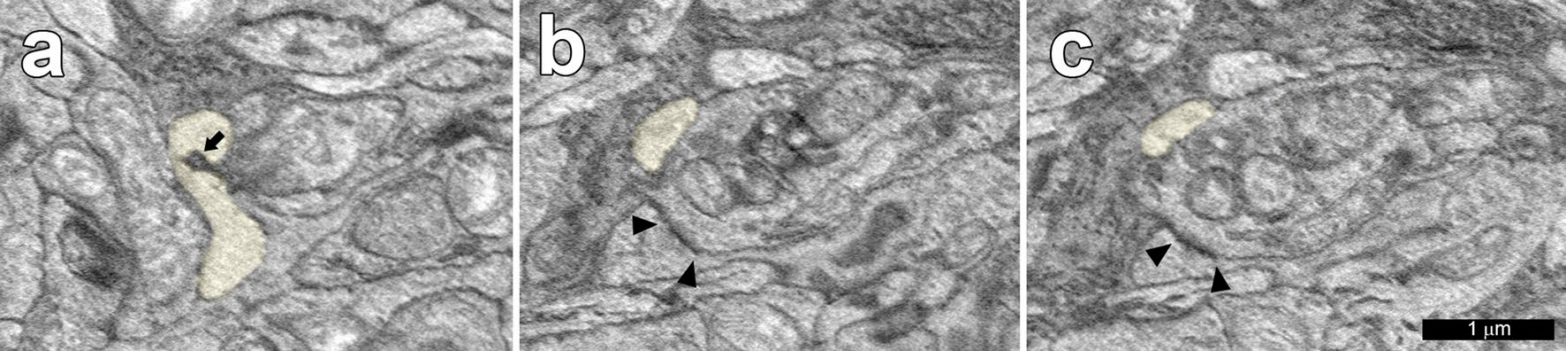
(**a**) Trogocytosis of OFF midget ganglion cell 50772 by microglial cell 50635 (yellow) (**b**,**c**) are consecutive sections through a synapse (arrowheads) onto the ganglion cell. (**a**,**b**) Separated by 4 70 nm horizontal sections.

Another major finding was that synapses, themselves, were never trogocytosed by microglia. In a few places, synapses were close to sites of trogocytosis, but they appeared to be unaffected by the microglial cells (Table 2). An example is illustrated in Fig. 5. One caveat is that the outer plexiform layer was not included in the volume we analyzed, and the functions of the microglia there are known to be different^24^. Another is that this tissue was collected in the middle of the day, and therefore we cannot rule out the possibility that synaptic pruning takes place at other times of the day using this dataset.

**Table 2:** Synapse distances from sites of trogocytosis.

Microglial cell ID	1				
Ganglion cell ID	47485	118	50584	223	50604
Distance in μm	0.24	0.22	0.43	0.27	0.33
Microglial cell ID	50635				
Ganglion cell ID	50763	50772			
Distance in μm	0.08	0.36			
Microglial cell ID	50636				
Ganglion cell ID	50904	59897			
Distance in μm	0.29	0.53			
Microglial cell ID	50640				
Ganglion cell ID	50923				
Distance in μm	0.18				
Microglial cell ID	50688				
Ganglion cell ID	50948	60957			
Distance in μm	0.50	0.09			

The geometric distances between sites of trogocytosis by microglial cells and the sites of the nearest synapses were calculated. OFF midget ganglion cells were selected for this analysis because their distal dendrites receive frequent synaptic inputs. This data provides support for our observation that sites of trogocytosis were sometimes close to sites of synapses but did not include the synapses, themselves.

## Discussion

The major finding of this study was that microglia in the IPL of the adult macaque retina engulf small portions of all the major types of neurons ramifying there. Based on the size of the engulfed elements, this interaction was defined as trogocytosis. This has also been observed in a study of the roles of microglia in pruning of synapses in the mouse hippocampus^25^. Presynaptic elements and axons, but not the synapses themselves, were selectively trogocytosed there. The trogocytosis was different in some respects in the retina than in the hippocampus. Unlike the hippocampus, presynaptic retinal neurons were not trogocytosed selectively. Retinal ganglion cell dendrites were always postsynaptic in the IPL, but small pieces of their dendrites were frequently engulfed. Müller glial cells were also trogocytosed by retinal microglia, albeit less frequently than the neurons.

The differences between our findings in macaque retina and those from the mouse hippocampus may be attributable to differences between microglia in the brain and the retina. There may also be differences the between microglia of rodents and primates. Both findings have been reported in a recent study of gene expression by brain and retinal microglia^26^. Another difference is that our study of macaque retina was done in an adult. The study of trogocytosis in the mouse hippocampus was done at postnatal day 15, when the mice were immature and synaptic remodeling was at its peak.

Because microglia play an important role in the elimination of synapses in the brain^27^, we had expected to see trogocytosis at retinal synapses. But there was no apparent selectivity for sites of retinal synapses. In those rare instances where synapses were located close to sites of trogocytosis, the synapses, themselves, appeared to be unaffected. However, because we used only electron microscopy in this study, we cannot rule out the possibility that microglia play a role in synaptic remodeling that is not detectable using this technique.

Another possible explanation for the trogocytosis of retinal neurons and glia by ramified microglia is that it prepares them to present retinal antigens to regulatory T cells when they become activated. Retinal microglia are not specialized for antigen presentation, and unlike other microglia in the eye, they do not become activated in response to systemic inflammation^28^. Under other conditions, however, retinal microglia do present antigens. In mouse models of Leber’s congenital amaurosis and experimental autoimmune uveoretinitis, retinal microglia present antigens^29,30^. In a single cell transcriptomic study of mice with autoimmune uveoretinitis, changes in gene expression by retinal microglia suggest that they also become capable of antigen presentation under those conditions^31^. In a rat model of traumatic optic neuropathy, injury-activated lymphocytes promoted the survival of retinal ganglion cells, but lymphocytes from uninjured control animals were cytotoxic. This neuroprotective effect required interactions between CD4^+^CD25^+^ regulatory T cells and activated retinal microglia^32^. Taken together, these findings suggest that antigen presentation to regulatory T cells by activated microglia would protect neurons and Müller glia when the blood retinal barrier is impaired and circulating immune cells infiltrate the retina. This interaction between regulatory T cells and activated microglia would be expected to have neuroprotective effects in healthy adult retinas.

## Supplementary Information

Supplementary Figures.

Supplementary Legends.

## Data Availability

The dataset analyzed during the current study is available to the public on a read-only basis (http://connectomes.utah.edu); the volume address is: http://v2486.host.s.uw.edu/Neitz/TemporalMonkey2/SliceToVolume.VikingXML. The data and codes used to generate figures are available upon request to the corresponding author. Figures were prepared using Adobe Photoshop CS6 and SBFSEM-tools. The code and data used to generate the figures in this study will be made available upon request. The Supplemental Figure was created using GIMP 2.10.32 (revision 1, www.gimp.org).

## References

[1] Silverman SM, Wong WT (2018). Microglia in the retina: Roles in development, maturity, and disease. Annu. Rev. Vis. Sci..

[2] Verkhratsky A, Sun D, Tanaka J (2021). Snapshot of microglial physiological functions. Neurochem. Int..

[3] Fan W (2022). Retinal microglia: Functions and diseases. Immunology.

[4] Wang SK, Cepko CL (2022). Targeting microglia to treat degenerative eye diseases. Front. Immunol..

[5] Li Q, Barres BA (2018). Microglia and macrophages in brain homeostasis and disease. Nat. Rev. Immunol..

[6] Singaravelu J, Zhao L, Fariss RN, Nork TM, Wong WT (2017). Microglia in the primate macula: Specializations in microglial distribution and morphology with retinal position and with aging. Brain Struct. Funct..

[7] Lee JE, Liang KJ, Fariss RN, Wong WT (2008). Ex vivo dynamic imaging of retinal microglia using time-lapse confocal microscopy. Invest. Ophthalmol. Vis. Sci..

[8] Paques M (2010). In vivo observation of the locomotion of microglial cells in the retina. Glia.

[9] Fontainhas AM (2011). Microglial morphology and dynamic behavior is regulated by ionotropic glutamatergic and GABAergic neurotransmission. PLoS One.

[10] Wang X (2016). Requirement for microglia for the maintenance of synaptic function and integrity in the mature retina. J. Neurosci..

[11] Patterson SS (2019). An S-cone circuit for edge detection in the primate retina. Sci. Rep..

[12] Patterson SS (2020). Wide-field amacrine cell inputs to ON parasol ganglion cells in macaque retina. J. Comput. Neurol..

[13] Bordt AS (2021). Synaptic inputs to broad thorny ganglion cells in macaque retina. J. Comput. Neurol..

[14] Anderson JR (2011). The Viking viewer for connectomics: Scalable multi-user annotation and summarization of large volume data sets. J. Microsc..

[15] Dowling JE, Boycott BB (1966). Organization of the primate retina: Electron microscopy. Proc. R. Soc. Lond. B Biol. Sci..

[16] Tsukamoto Y, Omi N (2015). OFF bipolar cells in macaque retina: Type-specific connectivity in the outer and inner synaptic layers. Front. Neuroanat..

[17] Tsukamoto Y, Omi N (2016). ON bipolar cells in macaque retina: Type-specific synaptic connectivity with special reference to OFF counterparts. Front. Neuroanat..

[18] Bordt AS (2019). Synaptic inputs from identified bipolar and amacrine cells to a sparsely branched ganglion cell in rabbit retina. Vis. Neurosci..

[19] Uribe-Querol E, Rosales C (2021). The multiple roles of trogocytosis in immunity, the nervous system, and development. Biomed. Res. Int..

[20] Dhodapkar RM, Martell D, Hafler BP (2022). Glial-mediated neuroinflammatory mechanisms in age-related macular degeneration. Semin. Immunopathol..

[21] Kolb H, Linberg KA, Fisher SK (1992). Neurons of the human retina: A Golgi study. J. Comput. Neurol..

[22] Mariani AP (1990). Amacrine cells of the rhesus monkey retina. J. Comput. Neurol..

[23] Marshak DW (1989). Peptidergic neurons of the macaque monkey retina. Neurosci. Res. Suppl..

[24] O'Koren EG (2019). Microglial function is distinct in different anatomical locations during retinal homeostasis and degeneration. Immunity.

[25] Weinhard L (2018). Microglia remodel synapses by presynaptic trogocytosis and spine head filopodia induction. Nat. Commun..

[26] Wolf J (2022). In-depth molecular profiling specifies human retinal microglia identity. Front. Immunol..

[27] Wilton DK, Dissing-Olesen L, Stevens B (2019). Neuron-glia signaling in synapse elimination. Annu. Rev. Neurosci..

[28] Dando SJ, Kazanis R, McMenamin PG (2021). Myeloid cells in the mouse retina and uveal tract respond differently to systemic inflammatory stimuli. Invest. Ophthalmol. Vis. Sci..

[29] Heuss ND, Lehmann U, Norbury CC, McPherson SW, Gregerson DS (2012). Local activation of dendritic cells alters the pathogenesis of autoimmune disease in the retina. J. Immunol..

[30] Tang PH, Pierson MJ, Heuss ND, Gregerson DS (2017). A subpopulation of activated retinal macrophages selectively migrated to regions of cone photoreceptor stress, but had limited effect on cone death in a mouse model for type 2 Leber congenital amaurosis. Mol. Cell Neurosci..

[31] Heng JS (2019). Comprehensive analysis of a mouse model of spontaneous uveoretinitis using single-cell RNA sequencing. Proc. Natl. Acad. Sci. USA.

[32] Geng Y, Lu Z, Guan J, van Rooijen N, Zhi Y (2021). Microglia/macrophages and CD4(+)CD25(+) T cells enhance the ability of injury-activated lymphocytes to reduce traumatic optic neuropathy in vitro. Front. Immunol..

