# Supplementary figures and images for: Trogocytosis of neurons and glial cells by microglia in a healthy adult macaque retina
Source: Sci Rep. 2023 Jan 12;13:633. doi: 10.1038/s41598-023-27453-2 (PMC9837165; doi:10.1038/s41598-023-27453-2)

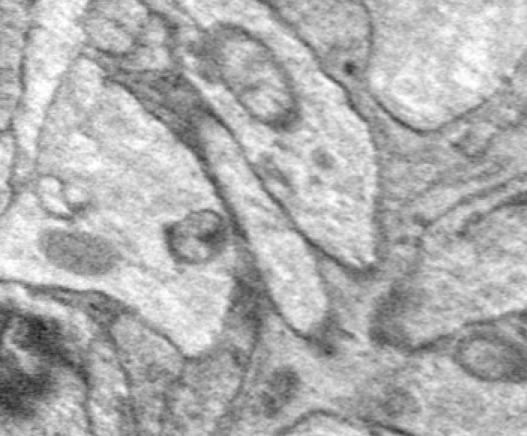

Supplement: Supplementary file 1 — Supplementary Figures. [file 41598_2023_27453_MOESM1_ESM.gif]
