# Supplementary material for: Trogocytosis of neurons and glial cells by microglia in a healthy adult macaque retina
Source: Sci Rep. 2023 Jan 12;13:633. doi: 10.1038/s41598-023-27453-2 (PMC9837165; doi:10.1038/s41598-023-27453-2)
Supplement: Supplementary file 2 — Supplementary Legends. [file 41598_2023_27453_MOESM2_ESM.docx]

Supplemental Figure. Trogocytosis by microglial cell 50635 of OFF midget ganglion cell 50772 (16 sections). Note the distance between the site of trogocytosis and the site of the synapse.
